# Linear predictive coding representation of correlated mutation for protein sequence alignment

**DOI:** 10.1186/1471-2105-11-S2-S2

**Published:** 2010-04-16

**Authors:** Chan-seok Jeong, Dongsup Kim

**Affiliations:** 1 Department of Bio and Brain Engineering, KAIST, 373-1 Guseong-dong, Yuseong-gu, Daejeon, 305-701, Korea

## Abstract

**Background:**

Although both conservation and correlated mutation (CM) are important information reflecting the different sorts of context in multiple sequence alignment, most of alignment methods use sequence profiles that only represent conservation. There is no general way to represent correlated mutation and incorporate it with sequence alignment yet.

**Methods:**

We develop a novel method, CM profile, to represent correlated mutation as the spectral feature derived by using linear predictive coding where correlated mutations among different positions are represented by a fixed number of values. We combine CM profile with conventional sequence profile to improve alignment quality.

**Results:**

For distantly related protein pairs, using CM profile improves the profile-profile alignment with or without predicted secondary structure. Especially, at superfamily level, combining CM profile with sequence profile improves profile-profile alignment by 9.5% while predicted secondary structure does by 6.0%. More significantly, using both of them improves profile-profile alignment by 13.9%. We also exemplify the effectiveness of CM profile by demonstrating that the resulting alignment preserves share coevolution and contacts.

**Conclusions:**

In this work, we introduce a novel method, CM profile, which represents correlated mutation information as paralleled form, and apply it to the protein sequence alignment problem. When combined with conventional sequence profile, CM profile improves alignment quality significantly better than predicted secondary structure information, which should be beneficial for target-template alignment in protein structure prediction. Because of the generality of CM profile, it can be used for other bioinformatics applications in the same way of using sequence profile.

## Background

Currently, the comparison of multiple sequence alignments (MSAs) is based on aligning the sequence profiles that represent conservation at specific positions. However, the alignment quality of profile-profile alignment becomes unreliable as the sequence identity of seed sequences becomes low [1]. Even though using predicted secondary structure as additional information slightly improves profile-profile alignment, it is still unsatisfactory since the most protein secondary structure prediction methods are based on sequence profile. In this situation, using correlated mutation information originated from coevolution of two or more residue positions would be informative.

Constructing alignments with high quality is important in comparative modeling, in which target-template alignment is a crucial step together with template selection, but the sequence alignments based solely on statistical amino acid matches become undependable at low sequence identity. Particularly, below 20% sequence identity referred to as midnight zone, using sequence alignment without structural evidence can be problematic. Practically, it is found that many proteins with similar structure have low sequence identity [2], and, in CASP7, about half of the targets have the single best templates with <20% sequence identity [3]. This means that the reliability of alignment in the midnight zone is a bottleneck for protein structure prediction, and therefore its improvement is strongly desirable.

Correlated mutation is estimated in various ways. McBASC algorithm [4] calculates the correlation of amino acid substitutions at individual positions. SCA algorithm and its variants [5-7] measure the relative amino acid frequencies observed after perturbing the MSA. Mutual information [8,9] is used for estimating correlated mutation. Recently, it is also found that normalizing mutual information improves the determination of coevolving residues [10,11]. In spite of these efforts, the application of correlated mutation is restricted mainly to inter-residue contact prediction [12,13] and functional site prediction [5]. Moreover, it has not been utilized for the purpose of sequence alignment, the most basic procedure in sequence analysis, and there is no universal method for comparing correlated mutation patterns of different proteins.

In this article, we introduce a novel method, CM profile, which represents correlated mutation based on signal processing technique called linear predictive coding (LPC) [14], and apply it to the protein sequence alignment problem. The results show that the employment of correlated mutation improves alignment quality consistently at different SCOP levels and sequence identities. The analysis on a few examples shows that the use of CM profile makes alignments preserve correlated mutation and the residues with common contacts are aligned with high scores.

## Methods

### Data

We prepare protein pairs which are non-redundant and distantly related with each other. The data are derived from SCOP [15] version 1.69 with <35% sequence identity downloaded from Astral compendium [16]. 4253 domains whose MSA is composed of less than 100 sequences are omitted because correlated mutation analysis using MSA with a small number of sequences can be unreliable and include much noise, and 2501 domains remain. To make pairs of distantly related homologs, we select superfamilies with at least 10 domains, and pair the domains with each other in each superfamily. The selected domains are composed of 1105 domains of 50 folds, 60 superfamilies, and 341 families. For parameter selection we use 388 pairs consisting of 200 domains randomly chosen, and for testing use 9118 pairs consisting of the remaining 905 domains.

The frequency matrices and the position-specific score matrices (PSSMs) representing sequence profiles are automatically generated by running PSI-BLAST [17] version 2.2.19 against NCBI *nr* database with “–j 3 –e 0.001 –h 0.001” options. The MSAs used for constructing CM profiles are also generated by running PSI-BLAST with the same option, and then thinned by removing the sequences covering less than 50% of the seed sequence and clustering the remaining sequences at 65% sequence identity.

### Representation of correlated mutation

If we have a sequence of length *n*, we build, at each position, 400 correlated mutation vectors consisting of *n* correlated mutation scores with other positions for one of 400 possible amino acids pairs. The correlated mutation vector for amino acid pair *a, b* at position *i*, *m*(*a_i_*, *b*) = [*m*(*a_i_*, *b*_1_) *m*(*a_i_*, *b*_2_) … *m*(*a_i_*, *b_n_*)], consists of *n* log-odds scores defined as

,

where *f*(*a_i_*, *b_j_*) is the joint frequency of amino acid *a* at position *i* and amino acid *b* at position *j*, *f*(*a_i_*) is the marginal frequency calculated as , and *f*(*b_j_*) is the marginal frequency calculated as . The correlated mutation score *m*(*a_i_*, *b_j_*) of amino acid *a* at position *i* and amino acid *b* at position *j* has a positive value if they have positive correlation with each other, a negative value if they have negative correlation, or zero if they evolve independently. Because a small number of observations can make the joint frequency very noisy, we apply low number correction [10], thereby defining the joint frequency as

,

where *N*(*a_i_*, *b_j_*) is the number of observations of amino acid *a* at position *i* and amino acid *b* at position *j* in the MSA, and .

Since the dimension of correlated mutation vector is variable depending on sequence length, the correlated mutation vectors of distinct sequences are not paralleled. Therefore, we extract the spectral features, known as LPC cepstral coefficients, to represent the correlated mutation vector. LPC cepstral coefficients have been used for comparing DNA and protein sequences [18].

The basic assumption of LPC model is that the present sample at time *n*, *s*(*n*), can be calculated as a linear combination of the past *p* samples. The approximation is expressed as [14]

,

where the coefficients {*a_k_*} are constants called as LPC coefficients, which are determined by minimizing the sum of squared error.

To solve this equation for the predictor coefficients {*a_k_*}, we differentiate *E* with respect to each *a_k_* and set the result to zero,.

The result gives a set of *p* linear equations

,

where *r*(*i*), known as the autocorrelation function of *s*(*n*), is defined as

,

and symmetric, i.e. *r*(–* k*) = *r*(*k*). The linear equations can be expressed in matrix form as

Since the *p* x *p* matrix of autocorrelation values is a Toeplitz matrix that is symmetric and all the diagonal elements are equal, the solution of the linear equations can be calculated recursively and very efficiently through Levinson-Durbin algorithm without relatively expensive computation such as matrix inversion. If the linear equation is solved, more advanced spectral feature called as LPC cepstral coefficients can be derived from the LPC coefficients by the following recursion.

By using the LPC analysis process described above, we transform a correlated mutation vector to the CM profile consisting of the LPC cepstral coefficients. Since the cepstral coefficients are decaying, we use only the first *L* coefficients excluding *c*_0_. Additionally, we normalize CM profiles of a protein by fitting the mean and variance into zero and one, respectively, to weight them equally regardless of the orders. We obtain consequently a *L*-dimensional CM profile, *c*(*a_i_*, *b*), that represents the correlated mutations between amino acid *a* at position *i* and amino acid *b* at other positions. In other word, all the correlated mutation between position *i* and other positions are represented as 400 x *L* coefficients.

### Alignment

To compare sequences, we define the alignment score between the position *i* of a protein and the position *j* of another protein as follows,

,

where *w_mut_*, *w_cor_*, and *w_sec_* denote the weights, and *S_mut_*(*i*, *j*), *S_cor_*(*i*, *j*), and *S_sec_*(*i*, *j*) denote the similarity scores of sequence profiles, CM profiles, and secondary structure predictions, respectively, between the positions *i* and *j*. *S_mut_*(*i*, *j*) is the sequence profile score defined as

,

where *q*(*a_i_*), *q*(*a_j_*), *t*(*a_i_*), and *t*(*a_j_*) are the frequencies and the PSSM scores of amino acid *a* at position *i* and *j* respectively. *S_cor_*(*i*, *j*) is the CM profile score defined as

,

Where *d*(*c*(*a_i_*, *b*), *c*(*a_j_*, *b*)) is the Euclidean distance between CM profile *c*(*a_i_*, *b*) at position *i* and CM profile *c*(*a_j_*, *b*) at position *j*, *d*_0_ is the threshold, and α is the scaling factor. The *S_cor_*(*i*, *j*) gives a positive score in case that the distance between CM profiles is less than *d*_0_, and a negative score in case that the distance is more than *d*_0_. *S_sec_*(*i*, *j*) is the secondary structure prediction score given as 1 if the predicted secondary structures at position *i* and *j* are identical, and 0 otherwise. We use PSIPRED [19] to predict secondary structures. Based on the score matrix consisting of *S*(*i*, *j*) for all *i* and *j*, we perform the Needleman-Wunsch algorithm with affine gap costs and baseline to find the optimal alignment.

### Assessment and parameter selection

We assess the alignment quality by measuring the average MaxSub score [20] of models derived from sequence alignments. The model is generated by directly copying the coordinates of C-alpha atoms based on the sequence alignment, and the MaxSub score of the model is computed with default options. The MaxSub score identifies the largest subset of C-alpha atoms of a model that superimpose well over the experimental structure, and provides a single normalized score in the range of 0 to 1. The MaxSub score 0 indicates a completely wrong model, and 1 indicates a perfect model. The parameters of each method are selected by simulated annealing (SA) that uses the average MaxSub score of training set as the objective function.

## Results

The selected parameters by simulated annealing are listed in Table 1. For *p* and *L* in LPC analysis, 6 and 9 are used, respectively. For α in CM profile score calculation, 0.025 is used. Combining CM profile, sequence profile, and secondary structure prediction (CMPA_PPA_SS) improves the conventional methods, the profile-profile alignment without and with secondary structure prediction (PPA and PPA_SS), by 7.6% and 4.7%, respectively. In addition, combining CM profile only with profile-profile alignment (CMPA_PPA) improves the PPA by 6.2%, which is 2.3 times more increase than PPA_SS which improves PPA by 2.7%. However, using CM profile solely performs poorly; therefore we will exclude it in the following test.

**Table 1:** Selected parameters for the different combination of scoring terms.

Method	Parameters							Average MaxSub
			
	*w_mut_*	*w_sec_*	*w_cor_*	*d*_0_	*g_open_*	*g_ext_*	*Base*	
PPA	1.0	-	-	-	6.0	0.5	0.5	0.3099
PPA_SS	1.0	1.5	-	-	5.0	0.5	0.0	0.3183
CMPA	-	-	1.0	2.8	7.0	1.7	0.5	0.2873
CMPA_PPA	1.0	-	0.5	3.2	8.0	0.4	1.0	0.3291
CMPA_PPA_SS	1.0	1.5	0.5	3.2	8.0	0.6	0.0	0.3334

*w_mut_*, *w_sec_*, *w_cor_*, and *d*_0_ denote the corresponding notations in alignment score function, and *g_open_*, *g_ext_*, and *base* denote gap-open cost, gap-extension cost, and baseline parameter used for dynamic programming procedure. PPA and PPA_SS denote profile-profile alignment without and with secondary structure prediction, respectively, CMPA denote alignment solely using CM profile, and CMPA_PPA and CMPA_PPA_SS denote alignment combining CM profile with PPA and PPA_SS, respectively.

As shown in Table 2, overall, CMPA_PPA_SS and CMPA_PPA significantly outperform the original methods, PPA_SS and PPA that do not use correlated mutation information by 4.1% (p-value 2.1e-252) and 5.0% (p-value 8.2e-232), respectively. The p-values are calculated by Wilcoxon signed rank test. At family level, using CM profile shows almost the same improvement that can be achieved by using secondary structure prediction. However, it is seen that CMPA_PPA_SS shows 2.2 times more improvement than PPA_SS and CMPA_PPA. A dramatic result can be seen at superfamily level where the average sequence identity is 11.5%, less than 17.4% at family level, and 96% of the protein pairs have sequence identity less than 20%. CMPA_PPA_SS and CMPA_PPA improve PPA by 13.9% and 9.5%, respectively, while PPA_SS does by 6.0%. This indicates that the use of correlated mutation is much more effective for difficult cases, where the sequence identity is very low, and the following analysis also consistently shows more effective improvements below 20% sequence identity. Moreover, it also implies that both the information can be used complementarily and many of the current alignment methods using secondary structure prediction can be improved by incorporating correlated mutation information. According to a previous study using data set derived from SCOP with <75% sequence identity, the sophisticated methods including the structural state assignment based on self-organizing map and the scoring function based on artificial neural network, have performed best and shown the average MaxSub score 0.22 at superfamily level [21]. In their work, the other methods have shown the average MaxSub scores of 0.20-0.22. The MaxSub scores of PPA and PPA_SS of this work are also in that range. Although the results cannot be directly compared, it is reasonable to expect that CMPA_PPA_SS should outperform the previous method on their test set as well.

**Table 2:** Average MaxSub scores of test set by different methods

Method	Average MaxSub		
	
	Family	Superfamily	All
PPA	0.4485	0.2053	0.2851
PPA_SS	0.4524	0.2177	0.2947
CMPA_PPA	0.4524	0.2248	0.2994
CMPA_PPA_SS	0.4572	0.2338	0.3070

Family and Superfamily denote the average MaxSub score at SCOP family and superfamily level, and All denotes the average MaxSub score of all samples. PPA and PPA_SS denote profile-profile alignment without and with secondary structure prediction, respectively, and CMPA_PPA and CMPA_PPA_SS denote alignment combining CM profile with PPA and PPA_SS, respectively.

**Figure 1:**
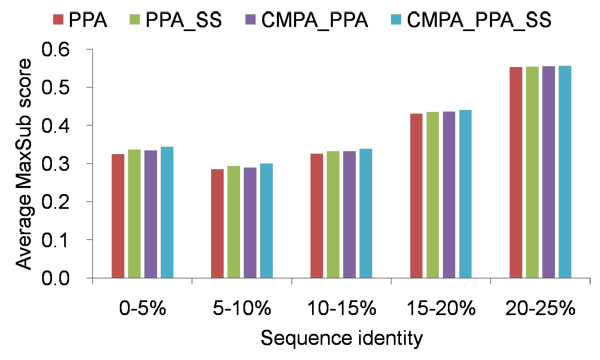
**Average MaxSub scores of various methods, measured by different sequence identities at family level** Average MaxSub scores above 25% sequence idenitty are not shown.

Figure 1 shows the average MaxSub scores of various methods at family level as a function of sequence identity. Below 20% sequence identity, CMPA_PPA_SS consistently outperforms the others and improves PPA by 3.2%. However, all the methods perform almost identically above 20% sequence identity. A reason for this result is that closely related proteins possibly share more common or similar sequences in their MSAs and yield similar sequence profiles. Particularly, the key residues strongly conserved are easily aligned and guide the global alignment optimally. According to a previous study [22], it has been demonstrated that the model quality generated by comparative modeling is related with the distribution of the sequence identity between the sequences comprised in the MSAs. Moreover, it is more probable that MSAs share more common or similar sequences, here, at family level where evolutionary distance is much closer than at superfamily level. Thus, in case where there is close evolutionary relationship and high sequence identity, sequence profiles seem to be sufficiently informative and combining correlated mutation or secondary structure prediction does not improve alignment quality.

**Figure 2:**
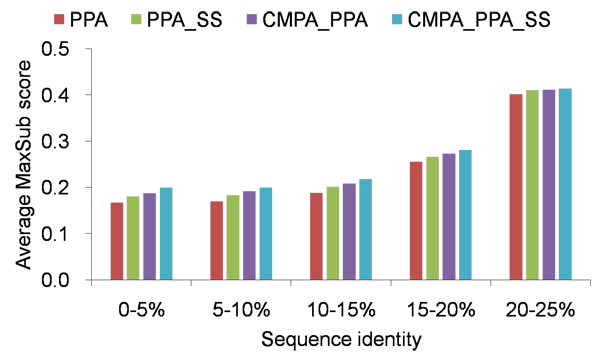
**Average MaxSub scores of various methods, measured by different sequence identities at superfamily level** Average MaxSub scores above 25% sequence idenitty are not shown.

As shown in Figure 2, CMPA_PPA_SS and CMPA_PPA outperform the conventional methods, PPA and PPA_SS, consistently, regardless of sequence identity at superfamily level. Similarly at family level, alignment quality deteriorates below 20% sequence identity, and the average MaxSub score of PPA does not exceed 0.19 below 15% sequence identity. Although CMPA_PPA_SS also shows a decline below 20% sequence identity, it significantly improves PPA and PPA_SS by 18.4% and 12.5%, respectively, in the range of 0-10% sequence identity, and by 13.0% and 8.9%, respectively, in the range of 10-20% sequence identity.

This result has important implications in several aspects. It is well known that profile-profile alignment is improved most by using secondary structure prediction [1,23] and numerous state-of-the-art methods hence incorporate secondary structure prediction in their alignment scheme [21,24-26]. Also in our results, profile-profile alignment is consistently outperformed by combining secondary structure prediction. However, it is more significantly improved by combining correlated mutation (CMPA_PPA), and the best performance is achieved by combining correlated mutation and secondary structure prediction together (CMPA_PPA_SS). Taking this into account, the state-of-the-art methods can be improved significantly by incorporating correlated mutation information.

Another aspect is related with the reliability of alignment in the midnight zone. An alignment becomes less reliable when the sequence identity lies in the midnight zone [1]. Since using correlated mutation is more advantageous for the proteins pairs with low sequence identity and CM profile is easily combined with conventional methods, the coverage of current alignment methods in the midnight zone can be increased by using CM profile. The most important implication is related with the template-based protein structure prediction. From two other aspects described above, it is obvious that using correlated mutation remarkably improves current alignment methods for the sequences with less than 20% sequence identity. This is beneficial to target-template alignment because most of promising templates sharing the same structure have relatively low sequence identity [2]. Practically, according to the recent analysis for the template-based modeling targets of CASP7 [3], almost half of the targets, specifically 50 among 108 targets, have the best templates with similar structure but low sequence identity less than 20%, and a virtual predictor based on the best templates overall outperforms all other groups by far. The effectiveness of CM profile will carry out more reliable target-template alignments and subsequently provide better models for difficult target-template pairs, thereby increasing the confidence for template-based structure prediction.

To assess the performance of CM profile for domains which have less MSA sequences than 100, we build two additional test sets from the omitted domains. The first test set is built from the domains with 50-99 MSA sequences, and consists of 527 domains of 30 folds, 31 superfamilies, and 142 families, deriving 5586 pairs. The second test set is built from the domains with 1-49 MSA sequences, and consists of 752 domains of 31 folds, 37 superfamilies, and 225 families, deriving 9676 pairs.

When testing with the domains whose MSA is composed of 50-99 sequences, using CM profile improves PPA and PPA_SS by 4.3% and 3.8%, respectively, as shown in Table 3. Also, when testing with the domains whose MSA is composed of 1-49 sequences, using CM profile improves PPA and PPA_SS by 2.4% and 3.5%, respectively, as shown in Table 4. Although the improvement rate becomes small for the domains with less MSA sequences, correlated mutation is still valuable as additional information for sequence alignment. Moreover, combining profile-profile alignment with both correlated mutation and secondary structure prediction outperforms the others significantly, convincing that both the information can complement each other. However, just as the quality of profile-profile alignment decrease when the amount of sequences in MSA is not sufficient, the performance of CM profile seems to decrease likewise.

**Table 3:** Average MaxSub scores of test set with 50-99 MSA sequences by different methods

Method	Average MaxSub		
	
	Family	Superfamily	All
PPA	0.4708	0.1714	0.2631
PPA_SS	0.4762	0.1839	0.2734
CMPA_PPA	0.4811	0.1832	0.2744
CMPA_PPA_SS	0.4852	0.1949	0.2838

Family and Superfamily denote the average MaxSub score at SCOP family and superfamily level, and All denotes the average MaxSub score of all samples. PPA and PPA_SS denote profile-profile alignment without and with secondary structure prediction, respectively, and CMPA_PPA and CMPA_PPA_SS denote alignment combining CM profile with PPA and PPA_SS, respectively.

**Table 4:** Average MaxSub scores of test set with 1-49 MSA sequences by different methods

Method	Average MaxSub		
	
	Family	Superfamily	All
PPA	0.4209	0.1816	0.2499
PPA_SS	0.4239	0.1925	0.2586
CMPA_PPA	0.4222	0.1896	0.2560
CMPA_PPA_SS	0.4305	0.2024	0.2676

Family and Superfamily denote the average MaxSub score at SCOP family and superfamily level, and All denotes the average MaxSub score of all samples. PPA and PPA_SS denote profile-profile alignment without and with secondary structure prediction, respectively, and CMPA_PPA and CMPA_PPA_SS denote alignment combining CM profile with PPA and PPA_SS, respectively.

## Discussion

The reason for the effectiveness of CM profile is related with the correlation between coevolution and contact. It has been shown that the residues important for protein function are not only conserved but also coevolved with other inter-related residues [12,27]. This fact has been exploited to infer the structural factor such as inter-residue contacts [13] and to evaluate the correctness of *de novo* model [28]. Recently, it has been also shown that key residues can be identified by analyzing residue-residue coevolution network [29]. In the aspect of alignment, contact-mutation matrices derived from structural information have been used for improving alignment quality [30]. CM profile utilizes correlated mutation information much more globally and progressively, implying all the correlated mutation of possible residue pairs. This optimizes alignment to match multiple contacting residue pairs, while the previous studies only consider at most two residue pairs, thereby improving alignment quality noticeably.

In the following, we exemplify that combining CM profile generates the alignment reflecting correlation mutation, and the aligned residues with high CM profile score are related with common contacts. As listed in Table 5, the proteins pairs are chosen from SCOP class a, b, c, and d, and share the same superfamily but different family.

**Table 5:** Protein pairs with the MaxSub scores of various methods

Protein 1 (SCOP classification)	Protein 2 (SCOP classification)	Sequence identity	PPA	PPA_SS	CMPA_PPA	CMPA_PPA_SS
d1xd7a_ (a.4.5.55)	d1ldja1 (a.4.5.34)	14.9	0.1197	0.1256	0.4042	0.4089
d1mvea_ (b.29.1.2)	d1ulea_ (b.29.1.3)	13	0.0515	0.0483	0.2201	0.2278
d1uwva2 (c.66.1.40)	d1p1ca_ (c.66.1.16)	9.4	0.0472	0.0533	0.2743	0.2996
d1lrza3 (d.108.1.4)	d1tiqa_ (d.108.1.1)	15.4	0.2458	0.2908	0.4420	0.4418

PPA and PPA_SS denote profile-profile alignment without and with secondary structure prediction, respectively, and CMPA_PPA and CMPA_PPA_SS denote alignment combining CM profile with PPA and PPA_SS, respectively.

**Figure 3:**
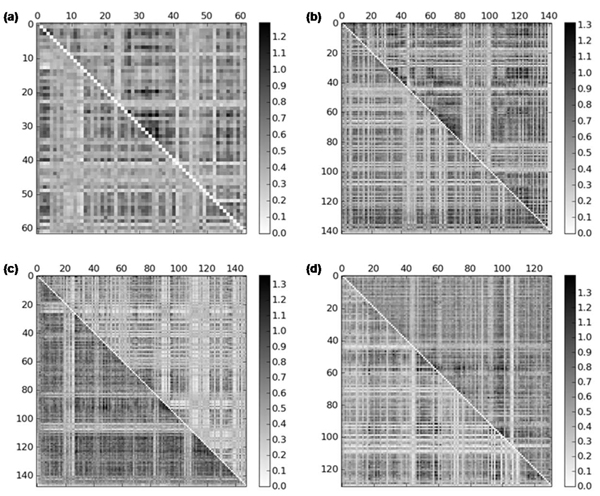
**Correlated mutation matrices of the proteins listed in Table **5 The upper-lower triangular matrix represents mutual information between the residues of the respective protein, (a) d1xd7a_-d1ldja1, (b) d1mvea_-d1u1ea_, (c) d1uwva2-d1p1ca_, and (d) d1lrza3-d1tiqa_, respectively. Note that the intensity and the size of image are scaled differently, regarding to the distribution of mutual information and the alignment length.

If the inter-related residues of a protein are aligned with the comparable residues of other protein, the correlated mutation information should be kept mutually in the resulted alignment. To demonstrate this, we investigate the correlation coefficient between correlated mutations of proteins. As correlated mutation measure, we calculate mutual information with low number correction [10], and the residues not aligned are excluded. The resulting correlated mutation scores for aligned residues are shown as matrix form in Figure 3. Although the matrices do not look completely symmetric, they have positive correlation coefficients, (a) 0.1208, (b) 0.1952, (c) 0.1131, and (d) 0.2118, respectively. These values are small, but all the correlations are statistically significant (p-value < 2.2e-308). In other words, the present method constructs an alignment that preserves coevolution.

**Figure 4:**
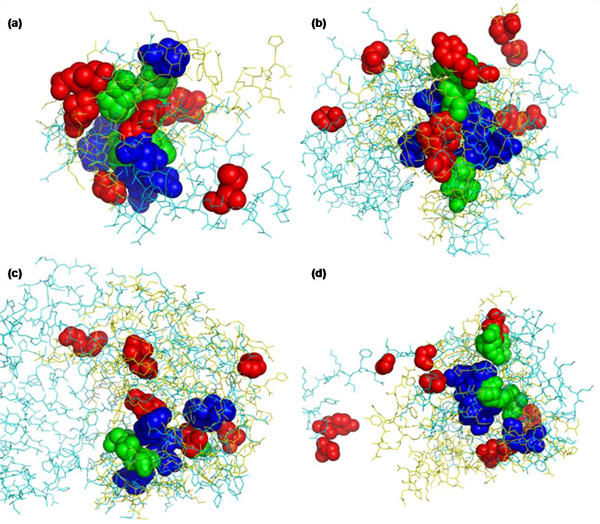
**High scoring residue pairs of the examples listed in Table 5** Each pair of proteins, (a) d1xd7a_-d1ldja1, (b) d1mvea_-d1u1ea_, (c) d1uwva2-d1p1ca_, and (d) d1lrza3-d1tiqa_ is superimposed based on the CMPA_PPA alignment, and coded as cyan-yellow, respectively. Top-10 residue pairs ranked by sequence profile score and CM profile score are shown as spheres coded as red and blue, respectively, but the residue pairs ranked by both are coded as green.

As shown in Figure 4, the residues with high CM profile scores are located spatially close with each other even though they are distant on sequence. On the other hand, the residues with high sequence profile scores are dispersed or not superimposed. This shows that CM profile reflects the structural aspect with regard to residue-residue contact in alignment and the common contacts of aligned proteins are ranked highly by CM profile score. This will be very useful in template-based protein structure prediction.

Due to the generality of CM profile, it can be successfully exploited for various bioinformatics applications, particularly with machine learning approaches. Our approach is position-specific and consists of a fixed number of values, which allows CM profile to be manipulated in the same way that we use sequence profile. Thus, CM profile can be easily adopted into the current methodology without serious modification to complement them. Moreover, it should be noted that our CM profile is not optimally generated because sequence profiles are automatically generated by PSI-BLAST [17]. The present method can be improved significantly, as the accuracy of correlated mutation is increased through various corrections and noise reductions [10,11].

## Conclusions

We develop a novel method to represent correlated mutation as the spectral features derived from LPC analysis, and we also apply it to sequence alignment of distantly related proteins. When combined with conventional sequence profile, CM profile improves alignment quality significantly better than predicted secondary structure information. Especially, the dramatic improvement in the midnight zone is observed, which should be beneficial for target-template alignment in protein structure prediction. Finally, because the methodology that we have developed in this work can be generalized to many interesting areas of bioinformatics, we expect that CM profile can be applicable to other bioinformatics applications equally well.

## Competing interests

The authors declare that they have no competing interests.

## Authors' contributions

CSJ designed the study, implemented the methods, performed the experiments and the result analysis, and drafted the manuscript. DK conceived of the study, participated in its design and coordination, and helped to draft the manuscript. All authors read and approved the final manuscript.
